# Mapping of angular leaf spot resistance QTL in common bean (*Phaseolus vulgaris* L.) under different environments

**DOI:** 10.1186/1471-2156-13-50

**Published:** 2012-06-27

**Authors:** Paula Rodrigues Oblessuc, Renata Moro Baroni, Antonio Augusto Franco Garcia, Alisson Fernando Chioratto, Sérgio Augusto Morais Carbonell, Luis Eduardo Aranha Camargo, Luciana Lasry Benchimol

**Affiliations:** 1 Departamento de Genética e Evolução e Bioagentes, Instituto de Biologia, Universidade Estadual de Campinas (UNICAMP), Campinas, SP, Brazil; 2 Instituto Agronômico (IAC), Av. Barão de Itapura 1481, CP 28, Campinas, São Paulo 13012-970, Brazil; 3 Universidade de São Paulo – Escola superior de Agricultura “Luiz de Queiroz” (ESALq/USP), Piracicaba, SP, Brazil

**Keywords:** *Phaseolus vulgaris* L., Angular leaf spot, Joint composite interval mapping, CIM, Resistance QTL

## Abstract

**Background:**

Common bean (*Phaseolus vulgaris* L.) is the most important grain legume for human diet worldwide and the angular leaf spot (ALS) is one of the most devastating diseases of this crop, leading to yield losses as high as 80%. In an attempt to breed resistant cultivars, it is important to first understand the inheritance mode of resistance and to develop tools that could be used in assisted breeding. Therefore, the aim of this study was to identify quantitative trait loci (QTL) controlling resistance to ALS under natural infection conditions in the field and under inoculated conditions in the greenhouse.

**Results:**

QTL analyses were made using phenotypic data from 346 recombinant inbreed lines from the IAC-UNA x CAL 143 cross, gathered in three experiments, two of which were conducted in the field in different seasons and one in the greenhouse. Joint composite interval mapping analysis of QTL x environment interaction was performed. In all, seven QTLs were mapped on five linkage groups. Most of them, with the exception of two, were significant in all experiments. Among these, ALS10.1^DG,UC^ presented major effects (R^2^ between 16% - 22%). This QTL was found linked to the GATS11b marker of linkage group B10, which was consistently amplified across a set of common bean lines and was associated with the resistance. Four new QTLs were identified. Between them the ALS5.2 showed an important effect (9.4%) under inoculated conditions in the greenhouse. ALS4.2 was another major QTL, under natural infection in the field, explaining 10.8% of the variability for resistance reaction. The other QTLs showed minor effects on resistance.

**Conclusions:**

The results indicated a quantitative inheritance pattern of ALS resistance in the common bean line CAL 143. QTL x environment interactions were observed. Moreover, the major QTL identified on linkage group B10 could be important for bean breeding, as it was stable in all the environments. Thereby, the GATS11b marker is a potential tool for marker assisted selection for ALS resistance.

## Background

The common bean (*Phaseolus vulgaris* L.) is an important source for human diet of protein, complex carbohydrates, fiber, isoflavones [1] and minerals such as iron and phosphorus [2]. This crop is cultivated in various countries around the world, among which Brazil stands out as the largest producer [3], with over 3,000 t produced in 2010 [4].

Several factors affect bean yield, among which the incidence of diseases is the biggest one. One of the diseases with the greatest impact is the angular leaf spot (ALS) [5,6]. The disease is caused by the fungus *Pseudocercospora griseola* (Sacc.) Crous & Braun (sin. *Phaeoisariopsis griseola* (Sacc.) Ferraris) [7], which causes necrotic lesions on the aerial parts of the plant, reducing the productivity and quality of the bean seed. Infection occurs due to conidia that penetrate through both the leaf epidermis and stomata, about three to seven days after inoculation [8]. It is a biotrophic fungus in the early stages of infection, which then becomes necrotrophic, when the attack causes the characteristic symptoms of the disease, which are angular necrotic spots limited by the leaf veins [9].

*P. griseola* presents great genetic variability and several physiological races [10-13] that can be grouped into two gene pools: Mesoamerican and Andean [14]. *P. griseola* isolates from the first group have a higher genetic variability [14] and infect both Andean and Mesoamerican bean cultivars, while isolates from the latter group infect bean plants only from the same origin [10].

Several sources of resistance to ALS have been identified [11,15,16] and among them, CAL 143 stands out due to having a high level of resistance against a large number of *P. griseola* races, whether in the field or the greenhouse [17]. This line is also resistant to rust, powdery mildew, alternaria leaf spot and anthracnose [18] and tolerant to variations of pH and low levels of phosphorus and nitrogen [19].

As the best form of disease control includes using resistant cultivars, the genetic characterization of resistance sources is very important for the genetic improvement of the crop. In the case of ALS, two dominant resistance genes have been described so far. The first, called *Phg-1*, was identified in the AND 277 variety [20] and recently mapped on linkage group B01 linked to markers from soybean [21]. The second, called *Phg-2*, was identified in the Mexico 54 variety [22] linked to SCAR OPN02 and RAPD OPE04 markers. The latter was recently mapped on linkage group B08 [23]. These two markers are linked to each other [24] and are also linked to the ALS resistance gene in the Cornell 49–242 [24], MAR 2 [25] and BAT 332 varieties [26]. Allelism tests showed that the *Phg-2* gene from Mexico 54 is the same as the BAT 332 gene [26]. Apart from these two genes, dominant monogenic inheritance for resistance to ALS has also been described in the Ouro Negro [27] and G10474 varieties [28], but the relationship of these genes with *Phg-1* and *Phg-2* remains unknown. Finally, there is also the case of US. Pinto 111, which presents recessive monogenic resistance [27].

In addition to qualitative resistance genes, there are also reports of QTLs controlling resistance to ALS. Five QTLs were mapped on linkage group B04, one on B08, one on B09 and three on linkage group B10 [23,29,30]. These studies revealed, therefore that resistance to ALS is more complex than described in the papers cited above. Mahuku et al. [23], for example, identified two resistance genes in G10909, where the gene mapped in the B08 group, and despite also being linked to the OPE04 marker, is distinct from *Phg-2*. These results substantiate the more complex inheritance theory towards resistance to ALS observed by Caixeta et al. [31]. These authors showed, via allelism tests, that three other genes (*Phg-3, Phg-4* and *Phg-5*), with two alleles each, control resistance to ALS in four bean varieties that were previously characterized as containing monogenic resistance (AND 277, Mexico 54, MAR 2 and Cornell 49–242).

The objective of this study was to identify QTLs that impart resistant to ALS by means of resistance quantitative analysis of 346 recombinant inbreed lines (RILs) derived from the IAC-UNA x CAL 143 (UC) cross. Line resistance was assessed in three experiments that reflect two distinct infection conditions: evaluation in the field (dry and wet seasons) under natural infection conditions, and evaluation in the greenhouse under controlled inoculation conditions. Linkage analysis between QTL and molecular markers, previously used to construct a genetic map for this same UC population [32], was carried out by means of joint composite interval mapping analysis (joint CIM; [33]).

## Results

### Statistical analysis of disease severity data

**Figure 1:**
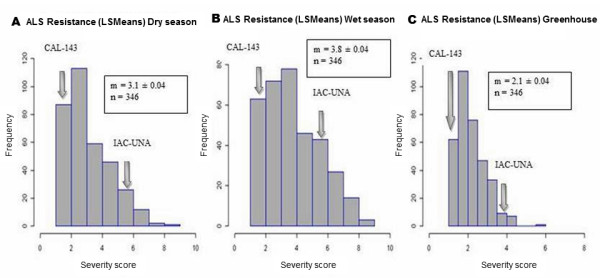
** Distribution of angular leaf spot (ALS) severity scores (Least Square Means - LSMeans).A**. Scores from RILs evaluated in the field during wet season and **B**. during dry season; **C**. severity scores from greenhouse experiments. Severity values of the parental lines (IAC-UNA and CAL 143) are indicated by arrows.

Angular necrotic spots, which are typical of the disease, were seen in the more susceptible RILs 10 days after inoculation on plants growing in the greenhouse and 30 days after sowing, in plants grown in the field. Parent lines presented a contrasting profile for resistance (Figure 1), as expected. The average severity for CAL 143 was 1.23 ± 0.4 considering all experiments, while for IAC-UNA, it was 5.16 ± 0.4 (Table 1). Broad-sense heritability for ALS resistance (Table 1) was high in both wet season experiment on field and in the greenhouse experiment and moderate in dry season experiment. Character showed high heritability (Table 1) in joint analysis.

**Table 1:** Means of disease severity values (LS Means) and heritability for each environment and joint analysis

**Genotype**	**Disease severity**			
	**Dry season**	**Wet season**	**Greenhouse**	**Joint**
**CAL-143**	1.0 ± 0.4	1.2 ± 0.4	1.5 ± 0.4	1.2 ± 0.4
**IAC-UNA**	5.6 ± 0.4	5.8 ± 0.4	4.1 ± 0.4	5.2 ± 0.4
**RILs**	3.1 ± 0.4	3.8 ± 0.4	2.2 ± 0.4	3.0 ± 0.4
**range**	1.0 – 8.1	1.1 – 8.9	1.0 – 5.6	1.0 – 8.9
**H**^**2**^	0.51	0.81	0.69	0.80

Standard errors for the parental lines and RILs; range of severity values observed on RILs and broad-sense heritability (H^2^) of angular leaf spot resistance, for each environment (dry and wet seasons field experiments and greenhouse) and joint analysis are indicated.

Severity levels among RILs ranged from 1.0 to 8.9 (Table 1). Severity data values of recombinant inbreed lines (RILs) showed normal distribution judging by skewness (0.0 for all experiments) and kurtosis values (0.32, 0.62 and 1.44 for dry and wet seasons and greenhouse experiments, respectively). Variance analyses (data not shown) confirmed great variability among RILs shown by highly significant values of F tests (F value 3.9, 2.4 and 1.82, p <0.0001 for dry and wet seasons and greenhouse experiments, respectively). Higher or lower resistance levels in relation to the parents were observed for RILs, which showed transgressive segregation.

The genetic and phenotypic correlations were significant in all pairwise combinations between experiments. Pearson’s (r_phe_) values were small, though significant (i.e. values different from zero with p-value ≤ 0.001), with lower values among field and greenhouse experiments (Table 2). The experiments showed no significant environmental correlation. This analysis also revealed a higher correlation between dry and wet seasons in field experiments in relation to the greenhouse experiment. As each of the three experiments proved to be a different environment, the QTL mapping analyses were not performed separately with the severity values for each experiment, but using a joint model [33]. The joint variance analysis revealed significant interaction between genotype x environment (F value 1.53, p <0.0001). 

**Table 2:** Pairwise correlation analysis between experiments: genetic, environmental and phenotypic correlation for the ALS severity values

**Environments**	**r**_gen_*	**r**_env_*	**Pearson’s Correlation (r**_phe_*)
**dry season x wet season**	0.841**	0.095	0.504**
**dry season x greenhouse**	0.685**	-0.038	0.334**
**wet season x greenhouse**	0.535**	0.009	0.339**

* r_gen_ = genetic correlation coefficient; r_env_ = environmental correlation coefficient; r_phe_ = Pearson’s correlation.

** significant at 1% based on *t* test (*p*-value ≤ 0.001).

### QTL mapping analysis

**Figure 2:**
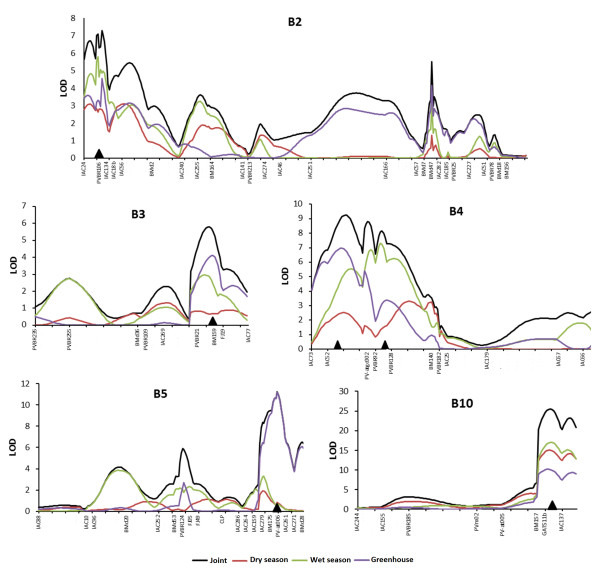
** QTL graphs indicating the LOD score values for each marker position.** LOD scores obtained by the joint CIM analysis (y) using the molecular makers distances of UC genetic map for each experiment (dry season – red; wet season – green and greenhouse – purple) and for the joint analysis (black). Black triangles indicate the position of maximum LOD values for significant QTLs. The linkage groups 1, 3, 4, 5 and 10 are indicated as B2, B3, B4, B5 and B10, respectively, according to Pedrosa et al. [36].

QTL mapping through joint composite interval mapping (joint CIM) analysis revealed seven QTLs (Table 3, Figure 2) which were named according to Miklas et al. [34]. Five QTLs were significant in all three experiments (ALS2.1^UC^, ALS4.1^GS,UC^, ALS4.2^GS,UC^, and ALS5.2^UC^ ALS10.1^DG,UC^; Figures 2A, C, E) and only two were not significant in dry season experiment (ALS3.1 and ALS5.1; Figures 2B and 2D, respectively). The ALS10.1 QTL, located on the B10 linkage group, showed the highest LOD and R^2^ values of all QTLs and the maximum LOD value was located on the same map position for all the experiments (Figure 2E). Two QTLs that were significant in all experiments showed greater effect only in one of them (Table 3): ALS4.2 had a greater effect in wet season experiment and ALS5.2 in the greenhouse. The remaining QTLs had minor effect on the phenotype. 

**Table 3:** Genetic parameters estimated by joint CIM analysis for angular leaf spot resistance

QTL*	**Linkage Group**	**Marker Interval**	**Environment**	**LOD**	**LOD Threshold**	**R**^**2**^**%**	**Additive effect**
**ALS2.1**^**UC**^	**B2**	**IAC134 -IAC18b**	**Joint**	**7.3**	**6.5**		
			dry season	2.8		2.2	-0.292
			wet season	5.8		5.6	-0.466
			gh**	4.5		1.9	-0.188
**ALS3.1**^**UC**^	**B3**	**PVBR21 - FJ19**	**Joint**	**5.8**	**3.4**		
			wet season	3.0		1.3	-0.344
			gh**	4.1		4.3	-0.165
**ALS4.1**^**GS,UC**^	**B4**	**IAC52 - BMd9**	**Joint**	**9.3**	**3.4**		
			dry season	2.5		1.4	-0.362
			wet season	5.5		0.7	-0.586
			gh**	7.0		4.4	-0.280
**ALS4.2**^**GS,UC**^	**B4**	**PVBR92 - Pv-gaat001**	**Joint**	**8.2**	**3.4**		
			dry season	3.3		0.8	-0.225
			wet season	7.3		10.8	-0.629
			gh**	3.4		2.0	-0.170
**ALS5.1**^**UC**^	**B5**	**BMd53 - FJ05**	**Joint**	**5.9**	**4.8**		
			wet season	2.3		2.9	-0.343
			gh**	2.7		0.2	0.149
**ALS5.2**^**UC**^	**B5**	**BM175 - IAC261**	**Joint**	**11.2**	**4.8**		
			dry season	1.9		1.3	-0.147
			wet season	3.3		1.3	-0.164
			gh**	11.2		9.4	-0.272
**ALS10.1**^**DG,UC**^	**B10**	**GATS11b - IAC137**	**Joint**	**25.5**	**3.5**		
			dry season	15.0		22.3	-0.703
			wet season	17.1		21.2	-0.913
			gh**	10.1		15.9	-0.304

*Resistance QTLs were named according to Miklas et al. [34]. ALS corresponding to disease name: Angular Leaf Spot; the first number refers to common bean linkage group; the second one is the QTL number for that linkage group in chronological order of publication; and superscript shorthand is the abbreviation for mapping populations used to discover each QTL, also in chronological order (GS = G5686 x Sprit [30]; GS = G10909 x Sprit [23]; DG = DOR364 x G19833 [29,35]; UC = IAC-UNA x CAL 143 [32]).

**gh = Greenhouse.

The percentage of phenotypic variation explained by the combined effects of each QTL was 28% in dry season field experiment, 42% in wet season field experiment and 38% in the greenhouse experiment. As expected, the values of additive effects of each QTL revealed that the alleles from the CAL 143 parent favored resistance in most loci, except in locus ALS5.1, whose favorable allele, in the greenhouse experiment, came from the susceptible parent IAC-UNA (Table 3).

### Major QTL validation across bean lines

The marker closest to the maximum LOD value from the major QTL ALS10.1 (GATS11b) was used to genotype resistant and susceptible bean lines. Only in two cases was there discrepancy between line phenotype and its genotype for the locus, where the BAT 332 and Mexico 54 lines, which are resistant to ALS, presented a marker allele from the susceptible parent (IAC-UNA). Thus, all susceptible lines presented the same allele as IAC-UNA, while three of five resistant lines showed the same allele as CAL 143 (Additional file 1).

## Discussion

This is the first QTL x environment interaction report for common bean resistance to ALS. The results revealed the existence of seven QTLs with variable magnitudes of phenotypic effects depending on the environments (Table 3), which indicated a complex and quantitative inheritance pattern of this trait on the CAL 143 line. The results contrast with those of other studies that reported dominant monogenic resistance inheritance [20,22,24,25,27,37,38]. One cause for this discrepancy is that these studies have assessed and analyzed the resistance in a qualitative manner rather than in a quantitative one. In fact, when using QTL analysis or when symptom evaluation is made quantitatively, different resistance genes may be observed in the same genotype. López et al. [29] conducted a QTL mapping through the DOR364 x G19833 population and found five ALS resistance QTLs. Mahuku et al. [23,30], on the other hand, used severity assessment on a quantitative scale and found three resistance genes in G5686 and two in G10909. Likewise, allelism tests showed that lines, previously characterized as containing dominant monogenic resistance, actually have different resistance loci to ALS, with allelic variations between lines [26,31]. It was shown, for example, that AND 277 has three other genes (*Phg-2*^*2*^ to *Phg-4*^*2*^) in addition to the previously identified *Phg-1*[31]. As AND 277 is one of CAL 143 parents, then it is possible that these three genes are segregating in the UC mapping population. The quantitative resistance nature to ALS can also be inferred by the presence of transgressive RILs both for resistance and susceptibility, a phenomenon observed both in field experiments as well as in the greenhouse.

A reasonable part of the phenotypic variation was explained by the sum of the effects of QTLs, especially in wet season and the greenhouse experiments. The lowest total of R^2^ observed in dry season may have occurred because the experiment was conducted during the dry season when the crop reaches the adult plant stage, since the dry climate discourages the development of the disease [8,9]. This condition did not prevail in wet season, which was carried out in the wet season, similar to the greenhouse, where conditions were controlled, with temperature and humidity favorable to fungal growth. These differences reflect the low correlations between the experiments probably due to genotype x environment interaction. However, the higher correlation between the field experiments than between them and the greenhouse was expected, since in the field the infection occurred in a natural way, differently of the experiment in the greenhouse.

Nevertheless, a major QTL (ALS10.1) was identified in all three experiments. This QTL is interesting because of its stability and its pronounced effect which explains the high resistance heritability revealed by variance analysis. A high heritability level for this trait was also reported by Amaro et al. [39] in a study of recurrent selection. The ALS10.1 QTL was located on linkage group B10, where López et al. [29] mapped a QTL with a large resistance effect for this same disease in the DOR364 × G19833 population. Due to being close to a resistance gene analog marker (RGA7) and to it also being linked to an anthracnose resistance gene, the authors suggested the existence of an R gene cluster in this genomic region. As RGA7 is linked to GATS11b (approximately 2 cM), it is very likely that the QTL reported by López et al. [29] corresponds to the ALS10.1 identified in this study.

The closest marker to the maximum LOD value in ALS10.1 (GATS11b) was used to validate this QTL in a set of lines that are resistant or susceptible to ALS. There was a correlation between phenotype and genotype marker in most cases. The two resistant genotypes (BAT 332 and Mexico 54) which presented the same marker allele as the susceptible IAC-UNA parent are known sources of Mesoamerican resistance, and due to this must have different resistance genes [31,38] which are not present in the GATS11b locus of ALS10.1. The hypothesis is reinforced by the fact that an allelism test with AND 277, CAL 143 parent, identified different genes in relation to Mexico 54, where the only gene in common (*Phg-2*) revealed a different allelic form [31]. Thus, it is plausible that the BAT 332 and Mexico 54 lines have a different allele for the ALS10.1 locus, taking into consideration that they are from diverging gene pools in relation to CAL 143.

QTLs with minor effects were also identified. Among these, ALS5.2 and ALS4.2 showed an interesting QTL x environment interaction. ALS5.2 revealed a greater resistance effect under greenhouse conditions, but only a small effect in the field experiments. ALS4.2 on the other hand, presented an opposite interaction with a greater resistance effect only under field conditions but not in the greenhouse. The remaining QTLs did not present such a variable effect among the experiments. Therefore, ALS4.2 and ALS5.2 are interesting QTLs for breeding approaches, as in the field the plants are subject to infection by different races of the pathogen, and in the greenhouse, the infection is race-specific, thus, the pyramiding of these two loci tends to result in more resistant cultivars in both conditions.

To date, no QTL has been identified on linkage group B05. The peak LOD score of the ALS5.2 QTL coincided with the position of the Pv-att006 marker in both individual and joint analysis. This is a microsatellite that occurs within a gene related to pathogenesis (PR gene) that codes for an endochitinase [40], which is an enzyme involved in the degradation of fungal cell walls. The co-localization between resistance QTLs and defense genes in plants reported in several pathosystems suggests the existence of a functional relationship between the QTLs and these genes [41]. The co-localization between an endochitinase and a resistance QTL, for example, has been reported in the pathosystem pepper - *Phytophthora capsici*[42].

The ALS4.1 and ALS4.2 QTLs were located on linkage group B04, where López et al. [29] reported resistance gene linkage to ALS with RGA markers. However, as there are no common markers on this linkage group, it was not possible to establish a relationship between resistance QTLs described by López et al. [29] and those mapped in this study. However, it is possible that ALS4.1 and ALS4.2 identified in this study and Phg_G5686A_ identified by Mahuku et al. [30] in the G5686 Andean line, are part of an Andean resistance gene cluster, as in the cross-map information [32,35], it can be noted that the Pv-ag004 marker (0.0 cM of Phg_G5686A_) is located between BMd 9 and PVBR92, that are close to the maximum LOD values for ALS4.1 (10 cM) and ALSb4.2 (4 cM).

Due to harboring genes that confer resistance to different *P. griseola* gene pool races, this cluster could be interesting to be used in common bean breeding programs, as the Andean resistance genes are most effective when transferred to cultivars of the Mesoamerican *pool* when they are grown in regions in which both Andean and Mesoamerican *P.griseola* isolates predominate [6,10]. Thus, the markers identified in this work in addition to those identified by Mahuku et al. [30] are applicable tools for marker assisted selection to obtain improved cultivars containing this ALS resistance cluster.

## Conclusion

The results indicate quantitative resistance control to angular leaf spot in CAL 143. Seven QTLs with variable effects were identified, four of which had never been mapped before. One major QTL of stable effect in the three experiments (ALS10.1) was identified in this study. The presence of this QTL explains the high heritability of the character reported in this study. Alleles at the GATS11b marker locus linked to the major QTL distinguished lines that are resistant and susceptible to ALS. Thus, GATS11b can be an important tool to be used in common bean improvement to carry out marker-assisted selection. The results also point to a significant QTL x environment interaction. ALS4.2 and ALS5.2 are seen as being interesting for application in bean breeding, since their pyramiding can lead towards obtaining more resistant cultivars under both infection conditions. In addition, the QTLs present on the B04 group could be part of a resistant gene cluster to different *P. griseola* gene pools*.*

## Methods

### Plant material

A population of 346 RILs in generation F_12_ from the IAC-UNA x CAL 143 cross [32] were used in the ALS resistance experiments. The IAC-UNA has Mesoamerican origin, is black seeded and susceptible to ALS, while CAL 143 has Andean origin, red striped calima type seeds and is resistant to ALS.

### Field evaluation of resistance to ALS

The RILs were evaluated in two experiments at the Experimental Station ‘Fazenda Santa Eliza’ (Instituto Agronômico in Campinas - SP, Brazil). The first was carried out in February/2009 during the dry season and the second in August/2009, in the wet season. The experimental plot consisted of a row with 10 plants/RIL, spaced 50 cm apart. The experimental design was a completely randomized block design with two replicates. The Carioca Comum cultivar was a susceptible genotype used as borderline. The IAC-UNA and CAL 143 parents and the Carioca Comum cultivar were also included as controls, between treatments.

The symptoms were evaluated approximately 60 days after planting, in the six central plants of the portions, using a diagrammatic scale that classifies the severity levels in grades ranging from 1 (no symptoms) to 9 (30% or more of leaf area with symptoms) [43].

### Greenhouse evaluation of resistance to ALS

The seeds were sown in plastic boxes (29.5 cm x 46.5 cm x 12.5 cm) with Dystrophic Red Latosol type soil, fertilized with NPK 04-14-08 (400 kg/ha), each containing 3 RILs sown in rows at a distance of approximately 4 cm, containing six plants each. The experimental design was also made up of completely randomized blocks with four replicates. The IAC-UNA and CAL 143 parents and the Carioca Comum cultivar were included among the treatments as controls.

Plants were inoculated 2 to 3 weeks after planting, when the plants were at the V3 development stage (first expanded trifoliate), by spraying both leaf surfaces with a 10^4^ conidia/ml suspension prepared from *P. griseola* monosporic colonies grown in V8 medium [8]. The isolate used (14259–2) was classified as belonging to the 0–39 race based on the reaction of the differential cultivars according to Pastor-Corrales et al. [11]. After inoculation, the RILs were kept for 48 h at room temperature between 22 to 24°C, relative humidity between 95 to 100% and photoperiod of 12 h [8]. After this period, plants were transferred to the greenhouse. The severity evaluation was made 17 days after inoculation, as described above.

### Statistical data analysis

Severity data were used for genetic (r_gen_), environmental (r_env_) and phenotypic (r_phe_) correlation analyses between experiments by the Genes software [44]. Spearman’s rank correlation as well as Pearson’s correlation were performed by the R software (packages "Hmisc" [45]). As the results for all phenotypic correlations were the same (data not shown), it was chosen the Pearson’s values to be discussed in the article. The severity data were also used in individual and joint variance analysis [46]. The normality of data distribution was evaluated by skweness and kurtosis values. Broad-sense heritability based on means was calculated for each experiment in joint analysis, using the mean square values of the ANOVAs [47]. Least Square Means (LSMeans) of severity of each RIL for each experiment/environment were used for QTL mapping.

### Joint CIM QTL mapping

Previously mapped microsatellite markers by Campos et al. [32], based on segregation data from UC population, were used to identify QTLs. The genetic map comprises 198 markers distributed into eleven bean linkage groups (B01 to B11), with a total length of 1865.9 cM and an average distance between markers of 9.4 cM. The joint composite interval mapping analysis (model 6 - JZmapqtl, [33]) was used to determine possible QTL x environment interactions. The QTL evidence was checked at 1 cM intervals and with a 10 cM window using the likehood ratio test (LRT). LRT values were converted to the LOD scale using formula: LOD = 0.2172 * LRT. The multiple regression (*stepwise*) with a 5% significance level was used to obtain the cofactors used in the CIM analysis, by the QTL Cartographer vs.1.17 software [48].

Due to the performance of multiple tests, the threshold values for QTL detection were determined separately for each linkage group, based on the Σi [(Ti/50) +1] formula, where Ti is the length in cM of the *ith* linkage group, considering adjacent regions every 50 cM as independent [49]. The threshold LOD values were compared to the maximum LOD values of the joint analysis for each linkage group to determine the presence of significant QTLs. The additive effect values were estimated for each experiment individually only for significant QTLs in the joint analysis. The model with significant QTLs for all linkage groups for each experiment was adjusted to determine the phenotypic variation (R^2^) explained by each QTL.

### Validation of marker linked to major QTL

A marker linked to the major QTL was used in the genotyping of 32 bean lines (Additional file 1). The line reactions to the 0–39 race were evaluated in the greenhouse as previously described. The symptom evaluation also followed the grade scale from 1–9 [43].

DNA extraction from each plant was performed according to Hoisington et al. [50]. Genotyping was conducted according to Campos et al. [32]. The polymorphic fragments were visualized on denaturing 6% polyacrylamide gels silver stained.

## Abbreviations

ALS: Angular Leaf Spot; ANOVA: Analysis of Variance; Joint CIM: Joint Composite Interval Mapping; LRT: Likehood Ratio Test; LSMeans: Least Square Means; QTL: Quantitative Trait Locus; RIL: Recombinant Inbreed Line; UC: IAC-UNA x CAL 143.

## Competing interests

The authors declare that they have no competing interests.

## Authors’ contributions

PRO conceived the experimental design; conducted the experiments and data analyses, and drafted the manuscript. RMB contributed with the genotyping and phenotyping, helping also on linkage and QTL analyses. LEAC participated in the initial design of the project, discussions and in the editing of the manuscript. AAFG supported QTL and experimental data. AFC and SAMC provided the mapping population and performed the ANOVA analyses. LLB conceived the project, was responsible for the project coordination, helped with data interpretation, and editing of the manuscript. All authors have read and approved the final version of the manuscript.

## Supplementary Material

Additional file 1 **Marker validation.** Common bean lines (*Phaseolus vulgaris* L.) used to validate the GATS11b marker, the closest linked marker to the maximum LOD value for the major QTL, ALS10.1. The resistance or susceptibility of each line to angular leaf spot is discriminated. Click here for file
