# Supplementary material for: Mapping of angular leaf spot resistance QTL in common bean (Phaseolus vulgaris L.) under different environments
Source: BMC Genet. 2012 Jun 27;13:50. doi: 10.1186/1471-2156-13-50 (PMC3464175; doi:10.1186/1471-2156-13-50)
Supplement: Additional file 1 — Marker validation. Common bean lines (Phaseolus vulgaris L.) used to validate the GATS11b marker, the closest linked marker to the maximum LOD value for the major QTL, ALS10.1. The resistance or susceptibility of each line to angular leaf spot is discriminated. [file 1471-2156-13-50-S1.docx]

**Additional data file**

**Supplement 1**. Common bean lines (*Phaseolus vulgaris* L.) used to validate the GATS11b marker, the closest linked marker to the maximum LOD value for the major QTL, ALS10.1. The resistance or susceptibility of each line to angular leaf spot is discriminated.

| **Common bean line** | **Institution Source*** | **GATS11b marker allele**** | **R/S***** |
| --- | --- | --- | --- |
| **SCS 202 Guará** | EPAGRI | B | S |
| **IAC - Alvorada** | IAC | B | S |
| **IAC - Apuã** | IAC | B | S |
| **IAC - Aysó** | IAC | B | S |
| **IAC - Carioca** | IAC | B | S |
| **IAC - Carioca Akytã** | IAC | B | S |
| **IAC - Carioca Aruã** | IAC | B | S |
| **IAC - Carioca Pyatã** | IAC | B | S |
| **IAC - Ybaté** | IAC | B | S |
| **IAPAR - 14** | IAPAR | B | S |
| **IAPAR - 57** | IAPAR | B | S |
| **IAPAR - 80** | IAPAR | B | S |
| **IAPAR - 72** | IAPAR | B | S |
| **IAPAR - 31** | IAPAR | B | S |
| **IPR - Aurora** | IAPAR | B | S |
| **Flor de Mayo** | CIAT | B | S |
| **L 507 - 1** | IAC | B | S |
| **ARC - 1** | CIAT | B | S |
| **Oito e Nove** | CIAT | B | S |
| **Alemão** | CIAT | B | S |
| **BAT - 93** | CIAT | B | S |
| **AB - 136** | CIAT | B | S |
| **OPS - 16** | UFLA | B | S |
| **Pérola** | EMBRAPA | B | S |
| **Z - 28** | UFLA | B | S |
| **PAN - 72** | CIAT | B | S |
| **IAC - UNA** | IAC | B | S |
| **CAL - 143** | CIAT | A | R |
| **Amendoim** | CIAT | A | R |
| **G5686** | CIAT | A | R |
| **BAT - 332** | CIAT | B | R |
| **México - 54** | CIAT | B | R |

* EPAGRI = Empresa de Pesquisa Agropecuária e Extensão Rural de Santa Catarina ; IAC = Instituto Agronômico do Estado de São Paulo; IAPAR = Instituto Agronômico do Estado do Paraná; CIAT = International Center for Tropical Agriculture; UFLA = Universidade Federal de Lavras; EMBRAPA = Brazilian Agricultural Research Corporation.

**A = marker allele of CAL 143; B = marker allele of IAC-UNA.

*** R/S = Resistant / Susceptible.
